# SLEEP DISTURBANCE AND DEPRESSIVE SYMPTOMS AMONG OLDER ADULTS: DO GENDER DIFFERENCES EXIST?

**DOI:** 10.1093/geroni/igad104.3085

**Published:** 2023-12-21

**Authors:** Suha You, Giyeon Kim

**Affiliations:** Chung-Ang University, Seoul, Republic of Korea; Chung-Ang University, Seoul, Republic of Korea

## Abstract

Sleep is a key part of a healthy lifestyle, but aging causes sleep problems by decreased melatonin levels. Sleep disturbance includes problems falling or staying asleep, too much sleep, or obstructive sleep apnea (OSA), the most common sleep-related breathing disorder in older adults. Thus, aging-related changes in sleep should be studied in various aspects of elderly’s health such as physical, mental, and cognitive health. As part of that, this study aims to (1) examine the connection between sleep disturbance and depressive symptoms and (2) investigate whether gender differences exist. Participants were Korean adults aged 65 and older (N=1,316) from the 2020 Korean National Health and Nutrition Examination Survey. The survey measured sleep disturbances (OSA, insomnia, hypersomnia), depressive symptoms, and sociodemographic characteristics. Hierarchical multiple regression analysis was conducted to examine whether sleep disturbances influence depressive symptoms and test the interaction between sleep disturbance and gender. Results revealed that after adjusting for covariates, those with more risk factors for OSA (B=1.056, p=.000) or with insomnia (B=1.527, p=.000) were more likely to have greater depressive symptoms than their counterparts. Additionally, the relative influences of OSA (B=1.295, p=.000) or insomnia (B=.965, p=.023) on depressive symptoms were contingent upon gender. However, no correlation was observed between hypersomnia and depressive symptoms. Although both OSA and insomnia are risk factors for depressive symptoms, older women with OSA or insomnia showed more severe depressive symptoms than older men with OSA or insomnia. Findings suggest the need to make sophisticated approaches considering homogeneity and heterogeneity within older adults.

